# Response of chloroplast pigments, sugars and phenolics of sweet cherry leaves to chilling

**DOI:** 10.1038/s41598-021-86732-y

**Published:** 2021-03-30

**Authors:** Matej Vosnjak, Helena Sircelj, Metka Hudina, Valentina Usenik

**Affiliations:** 1 Chair for Fruit Growing, Viticulture and Vegetable Growing, Department of Agronomy, Biotechnical Faculty, University of Ljubljana, Jamnikarjeva 101, 1000 Ljubljana, Slovenia; 2 Chair of Applied Botany, Plant Ecology & Physiology and Informatics, Department of Agronomy, Biotechnical Faculty, University of Ljubljana, Jamnikarjeva 101, 1000 Ljubljana, Slovenia

**Keywords:** Plant physiology, Secondary metabolism, Plant sciences, Plant stress responses, Abiotic, Biochemistry, Carbohydrates, Monosaccharides, Sugar alcohols, Physiology, Metabolism

## Abstract

The aim of the present study was to evaluate the effect of post-flowering chilling of sweet cherry (*Prunus avium* L.) on the content of biochemical parameters in the leaf (chloroplast pigments, sugars and phenolics). The effect of chilling was investigated in two experiments. Potted 2-year-old trees of cv. 'Grace Star' and 'Schneiders' were exposed to one, two or three consecutive overnight chillings at an average air temperature of 4.7 °C (Experiment I), but in the following year only trees of 'Grace Star' were chilled at 2.2 °C (Experiment II), 3 to 7 weeks after flowering. The analysis of the biochemical parameters was performed by high performance liquid chromatography combined with electrospray ionization mass spectrometry. Chilling at 4.7 °C caused little or no stress, while 2.2 °C induced more intense stress with increased zeaxanthin, sugar and phenolic content in leaves, while exposure of trees to higher temperatures and closer to flowering showed no changes. Two or three consecutive overnight chilling periods increased the phenolic content and enhanced the accumulation of zeaxanthin in the leaves. Sucrose, sorbitol, fructose, total sugar, and total flavonoid content in leaves increased within 48 h after chilling. Zeaxanthin epoxidized within 24 h after one and 48 h after one and two consecutive overnight chillings.

## Introduction

The effects of global warming are of great importance for the productivity of fruit crops in the temperate zone^1^. The sweet cherry (*Prunus avium* L.), an economically important and early-flowering stone fruit of temperate zone, has shown increasingly earlier flowering^2^. With its earlier flowering, the trees are more frequently exposed to temperature extremes under which the flowering and fruit development of the sweet cherry is at risk^3^. In temperate regions, low temperatures are the primary abiotic stress limiting plant productivity^4^. The low temperature that limits plant productivity above freezing point of 0 °C is called chilling stress^5,6^. The optimal temperature for sweet cherry growth is 25°C^7^, but 4.5 °C is the temperature threshold, below which sweet cherry growth is zero^8^. Any deviation from the optimal temperature leads to a number of disturbances in the growth and development of the plant. Although there is no visible damage to trees, chilling stress can trigger various molecular, biochemical and physiological changes in plants, together with the subsequent morphological symptoms referred to as chilling injury^9,10^, which could influence the further growth and development of the sweet cherry.

The stress induced by chilling varies with temperature and depends on the duration^9^, the plant species, the developmental stage of the plant and the conditions before and during chilling. The different sensitivity to chilling is related to the composition of membrane lipids and another antioxidant defense mechanism of plants^10^. Most studies reporting on the biochemical response to chilling stress focus on sensitive annual plant species, such as maize^11^, cucumber^12^, pepper^13^ and others. Few data have been found on the effects of chilling on the response of temperate fruit species, considered chilling more tolerant.

Chilling stress is associated with disturbances in physiology (including all essential components of photosynthesis)^5^, membrane integrity, gene expression, ion leakage, proteins and other biomolecule activities^9^. In response to chilling stress, free radicals and reactive oxygen species (ROS) are overproduced, resulting in oxidative damage to essential plant structures^14^. Under stress conditions, the primary metabolism of plants (e.g. sugars) acts as a direct indicator of photosynthetic performance^15^, while secondary metabolites (e.g. phenolics) react as antioxidants, ROS scavengers, coenzymes and as regulatory molecules^4^.

Under stress, sugars also play an important role in carbon storage by acting as signal molecules that modulate gene expression and partly scavenge ROS^15–17^. Various studies have shown an increase in sugar under chilling, such as in cucumber^12^, spinach^18^, etc. Sicher^17^ found higher sucrose, fructose and glucose levels in leaves of *Arabidopsis thaliana* after 24 h chilling at 6 °C.

To counteract the effects of chilling and thus oxidative damage, plants activate various antioxidative mechanisms for ROS scavenging, which can be either enzymatic or non-enzymatic. Together with ascorbic acid, tocopherols and glutathione, phenolics and carotenoids are important non-enzymatic antioxidant components^14^.

Chilling stress induces phenylpropanoid metabolism and activates flavonoid biosynthesis enzymes (phenylalanine ammonia-lyase enzyme)^4^, which leads to increased synthesis of phenolic compounds to survive the stress conditions^19^. Christie et al.^20^ reported the accumulation of anthocyanins in maize leaves exposed to 5 °C, 10 °C and 15 °C. Oh et al.^21^ and Cansev et al.^22^ found a higher total phenolic content in the leaves of *Lactuca sativa* (exposed at 4 °C for 1 day) and *Olea europaea* (exposed at 4 °C for 12 h).

Chilling stress affects the chloroplast pigments, both carotenoids and chlorophylls^13,23^. Carotenoids act as accessory light pigments, ROS scavengers and have photoprotective functions^14,24^. In response to stress, the pigments of the xanthophyll cycle, an important subgroup of carotenoids, play a dominant role. Under excess excitation energy that cannot be used for photosynthesis, zeaxanthin and antheraxanthin are formed from violaxanthin by the deepoxidation cycle to thermally dissipate the absorbed excitation energy^11^. An increased zeaxanthin content has already been reported as an early indicator of stress^25^. A decreased chlorophyll content is also typical for oxidative stress due to chilling, as has been shown for the coffee plant^26^ or *Vitis vinifera*^27^. In maize plants exposed to low temperatures an altered pigment composition with a reduction of β-carotene and an accumulation of zeaxanthin was reported^28^.

In the context of global warming, a better understanding of the temperature-related limitations of sweet cherry is essential, especially in regions exposed to warmer and more variable winters^3^, where flowering starts earlier. The aim of the present study was to investigate the effects of post-flowering chilling on the biochemical changes of sweet cherry leaves. An experiment was set up to answer the question whether (1) overnight chilling increases the content of chloroplast pigments, soluble sugars and phenolic compounds in sweet cherry leaves; (2) several consecutive overnight chillings affect the metabolite content differently than a single exposure; (3) several overnight chillings lead to a different recovery than a single chilling; (4) the cultivars differ in their biochemical response to overnight chilling.

## Materials and methods

### Plant material and chilling treatments

At the Biotechnical Faculty of the University of Ljubljana, Slovenia (46° 2′ N, 14° 28′ E, 297 m a.s.l), two experiments of the overnight exposure of sweet cherry (*Prunus avium* L.) trees to chilling temperatures (4.7 °C and 2.2 °C) were carried out. The trial started with 2-year-old trees in 76-l pots of two cultivars 'Grace Star' and 'Schneiders Späte Knorpelkirsche' (syn. 'Schneiders'), on a Gisela 5 rootstock in Experiment I and was continued with 3-year-old trees of 'Grace Star' in Experiment II. The plant material used in the study is freely available in all fruit tree nurseries. The collection of the plant material complied with relevant institutional, national and international guidelines and legislation. Formal identification of the plant material used in our study was carried out by the Chair of Fruit Growing, Viticulture and Vegetable Growing, Department of Agronomy, Biotechnical Faculty, University of Ljubljana. A voucher specimen of this material has not been deposited in a publicly available herbarium.

**Figure 1:**
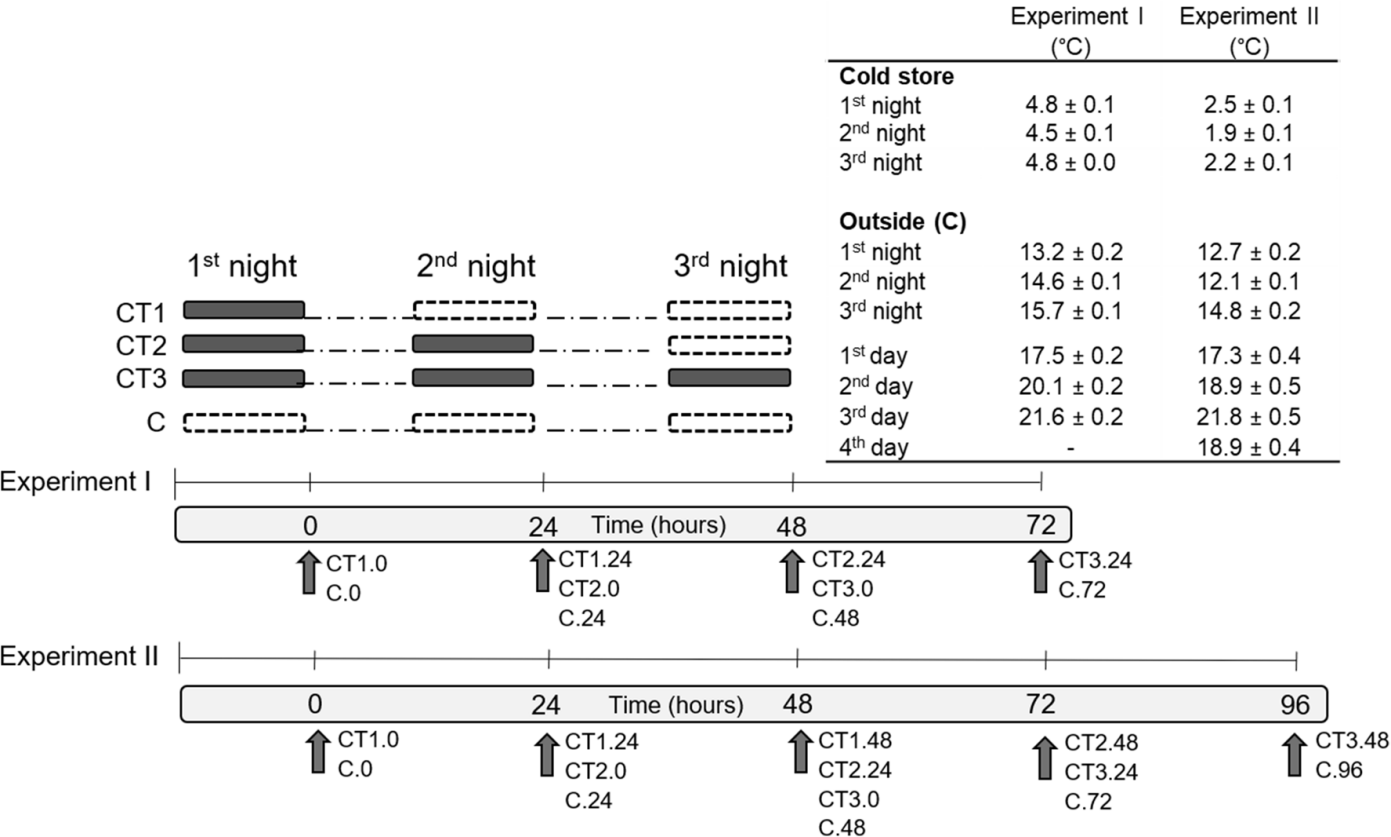
Illustration of the experimental set up (CT1, CT2, CT3, chilling treatments; C, control), the sampling procedure/time (hours) and temperatures (presented as mean ± SE for night-time temperatures in cold store and outside and daytime temperatures; °C) for experiments I and II. A black filled rectangle indicates the night-time exposure to chilling temperature in cold store, while white filled rectangle indicates the night under natural conditions (outside). The arrows indicate the sampling time, with the list of measured CT.time levels next to the arrows.

Eighteen trees of each cultivar were randomly arranged to trees exposed to chilling treatments (CT; 9 trees) and controls (C; 9 trees). Three CT patterns were determined (each comprising 3 trees). The CT1 trees were exposed to the chilling temperature in one night, the CT2 trees in two consecutive nights and the CT3 trees in three consecutive nights (Fig. 1).

The CT trees were placed in a cold store at sunset (8:30 pm) and transported outside at sunrise (5:30 am), to ensure the same photoperiod for CT trees and controls. The first night all CT trees were placed into a cold store, the second night only CT2 and CT3 trees and the third night only CT3 trees. The control trees were under natural conditions during the night (experimental setup in Fig. 1). During the day, all trees were exposed to the same environmental conditions. The daytime temperatures were in the optimal range for the period of the experiments I and II (see inset table in Fig. 1).

The night-time chilling temperatures differed between experiments I and II. Based on the results of Experiment I, the average temperature in Experiment II was reduced from about 4.7 °C to an average of 2.2 °C. The control trees in experiments I and II were exposed to a higher average air temperature during the night (on average 9.8 °C and 11.0 °C) (Fig. 1). In Experiment I, the trees of ‘Grace Star' and ‘Schneiders' were exposed for the first time 27 and 21 days after flowering, but ‘Grace Star' in Experiment II 50 days after flowering.

### Chemicals

The following standards were used for identification of compounds: glucose, fructose, sucrose, sorbitol, quercetin-3-*O*-glucoside, kaempferol-3-*O*-glucoside, ( −)-epicatechin and ( +)-catechin from Fluka Chemie GmbH (Buchs, Switzerland); neochlorogenic (3-caffeoylquinic) acid, chlorogenic (5-caffeoylquinic) acid and quercetin-3-*O*-rutinoside from Sigma–Aldrich Chemical (St. Louis, MO, USA). Standards of chlorophyll a and b, neoxanthin, lutein, violaxanthin, antheraxanthin, zeaxanthin and β-carotene were from DHI LAB products (Hørsholm, Denmark). Methanol for the extraction of phenolics and acetone for the extraction of chloroplast pigments was acquired from Sigma–Aldrich Chemical. The chemicals for the mobile phases were HPLC–MS grade acetonitrile and formic acid from Fluka, acetone and ethyl acetate from Merck Chemical GmbH. Water for the mobile phase was double distilled and purified with the Milli-Q system (Millipore, Bedford, MA, USA).

### Sampling and sample preparation for biochemical analysis

Five fully developed and undamaged leaves per tree (CT and control) with similar exposure were randomly sampled at 6:00 solar time in the morning, when the trees were taken out of the cold store (time 0), to ensure equal irradiation conditions. To achieve a recovery response, sampling was repeated 24 h after (Experiment I; time 24) and also 48 h after each CT (Experiment II; time 48) (Fig. 1). The sampled leaves were immediately frozen in liquid nitrogen, lyophilized, ground to a fine powder in a cooled grinder and stored in humidity proof, dark plastic containers at -20 °C, until analysis.

### Extraction

Leaf samples were analyzed for the content of chloroplast pigments (experiments I and II), sugars and phenolics (Experiment II).

Chloroplast pigments were extracted according to Tausz et al.^29^. Briefly, 0.1 g lyophilized dry leaf powder was extracted with 3 mL of 100% ice-cold acetone. The samples were homogenized with an Ultra-Turrax homogenizer for 30 s. After homogenization the samples were centrifuged at 10,000 rpm and 4 °C for 10 min, filtered through Minisart (SRP 15, PTFE) polyamide filters (Sartorius Stedim Biotech, Göttingen, Germany) and transferred to vials. All extraction procedures were performed in dim light.

Sugars were extracted as reported by Usenik et al.^30^ as follows: 0.4 g lyophilized dry leaf powder was homogenized in 7 mL of double-distilled water and left for 30 min at room temperature with frequent stirring. The extracts were centrifuged at 10,000 rpm for 10 min at 4 °C (Eppendorf Centrifuge 5810R; Hamburg, Germany). After extraction, the supernatants were filtered through a 0.20 μm cellulose mixed ester Chromafil A-20/25 filter (Macherey–Nagel, Düren, Germany) and transferred to vials.

Phenolics were extracted as follows: 0.04 g of lyophilized dry leaf powder was homogenized in 7 mL 80% methanol with 3% formic acid (v/v) by vortexing and extracted in a cooled ultrasonic bath for 1 h. After extraction, the samples were centrifuged at 10,000 rpm and 4 °C for 10 min, filtered through Chromafil AO-20/25 polyamide filters (Macherey–Nagel, Düren, Germany) and transferred to vials^30^.

### HPLC analysis

Chloroplast pigments (chlorophylls and carotenoids) were determined using the method described and cited in Tausz et al.^29^. Briefly, pigments were analyzed by HPLC–DAD (Thermo Finnigan, San Jose, California, USA) and a column Spherisorb S5 ODS-2 (250 × 4.6 mm) with an S5 ODS-2 (50 × 4.6 mm) precolumn (Alltech Associaties, Inc., Deerfield, Illinois, USA). The solvent gradient was from 10% B to 75% B in the first 18 min, then to 70% B in 7 min, to 100% B in next 5 min, and returning to the initial conditions in 2 min. Mobile phase A was acetonitrile, water and methanol (100/10/5; v/v/v) and mobile phase B was acetone with ethyl acetate (2/1; v/v). Flow rate was maintained at 1 mL/min. Detection of chloroplast pigments was performed at 440 nm. The quantification of identified compounds was based on peak area and expressed as mg/g dry weight (DW).

The analysis of sugars was performed according to Usenik et al.^30^ on a Surveyor HPLC system with a refractive index (RI) detector (Thermo Scientific, Finnigan Spectra system, Waltham, MA, USA). Separation was carried out using Rezex-RCM-monosaccharide Ca^+^ (2%) column (300 mm × 7.8 mm; Phenomenex, Torrance, CA, USA), operated at 65 °C. The elution solvent was double distilled water. The injection volume was 20 μL, flow rate 0.6 mL/min, and the run time 30 min. The quantification of identified compounds was based on peak area and expressed in mg/g DW.

The analysis of phenolic compounds was performed with a Dionex UltiMate 3000 HPLC system (Thermo Scientific, San Jose, California, USA) system, with absorbance monitored at 280 (hydroxycinnamic acids) and 350 nm (flavonoids). The separation was performed on a Gemini C18 (150 × 4.6 mm 3 µm, Phenomenex, Torrance, CA, USA) column at 25 °C. Mobile phase A was 3% acetonitrile with 0.1% formic acid in double distilled water (v/v/v). Mobile phase B was 3% double distilled water with 0.1% formic acid in acetonitrile (v/v/v). The following linear gradient was used: 0–15 min, 5% solvent B; 15–20 min, 20% B; 20–30 min, 30% B; 30–35 min, 90% B; and 35–45 min, 100% B before returning to the initial conditions to the end of the run time (50 min). The injection volume was 20 µL and the flow rate 0.6 mL/min^30^. The identification of phenolic compounds was confirmed by spectra characteristics, comparing retention times and using LCQ Deca XP MAX mass spectrometer (Thermo Finnigan, San Jose, California, USA) with an electrospray ionization (ESI) operating in negative ion mode. The content of individual compound was calculated from calibration curves of the corresponding standard and expressed in mg/g DW. The quantification of compounds for which no standards were available was performed with similar compounds; 3,5-di-*O*-caffeoylquinic acid as the equivalent of chlorogenic acid and quercetin-diglucoside as the equivalent of quercetin-3-*O*-glucoside.

The sum of all compounds from the specific group identified in the study was calculated as follows: total sugars (sum of glucose, fructose, sucrose and sorbitol), total hydroxycinnamic acids (sum of neochlorogenic acid, chlorogenic acid and 3,5-di-*O*-caffeoylquinic acid), total flavonoids (sum of quercetin-3-*O*-rutinoside, quercetin-3-*O*-glucoside, quercetin-diglucoside, kaempferol-3-*O*-glucoside, catechin and epicatechin), chlorophyll a/b (ratio of chlorophyll a and chlorophyll b), total chlorophylls (sum of chlorophyll a and chlorophyll b), total carotenoids (sum of lutein, β-carotene, neoxanthin, violaxanthin (V), antheraxanthin (A) and zeaxanthin (Z)) and xanthophyll cycle pigments (VAZ) (sum of violaxanthin, antheraxanthin and zeaxanthin). Deepoxidation state of xanthophyll cycle pool (AZ/VAZ) was calculated as ratio of (A + Z) / (V + A + Z).

### Measurements of chlorophyll fluorescence parameters

The measurements of the maximum quantum yield of PS II (Fv/Fm) were performed on one leaf per tree at about 6:00 solar time, when the trees were taken out of the cold store—when CT1, CT2 and CT3 had finished their exposure, by using the portable pulse amplitude modulated chlorophyll fluorometer PAM 2500 (Walz, Effeltrich, Germany). The leaves were first acclimated to dark conditions to ensure that all reaction centers were in the open state. To adapt to dark conditions, the leaves were kept in cuvettes for 30 min before measurement. The fluorescence was excited with a saturating irradiance of the ‘white light’ pulse (photosynthetic photon flux density, 8000 μmol m^−2^ s^−1^; 0.8 s)^31^.

### Statistical analysis

The statistical analysis was processed using the software version 3.6.1. of R statistical environment^32^. A different statistical approach was used for experiments I and II.

In Experiment I, the data were analyzed as a two‐factor-experiment with analysis of variance. The factor cultivar had two levels (‘Grace Star' and ‘Schneiders') and factor CT.time 10 levels (CT1.0, CT1.24, CT2.0, CT2.24, CT3.0, CT3.24, C.0, C.24, C.48 and C.72) for chloroplast pigments, while 5 levels for chlorophyll fluorescence parameters 5 levels (CT1.0, CT1.24, CT2.0, C.0 and C.24). Factor values were determined on the basis of CT treatments (CT1, CT2, CT3) with time after exposure (0 or 24 h) and controls (C) with time (0, 24, 48 and 72 h).

The data of the Experiment II were analyzed as one‐factor experiment with analysis of variance. The factor CT.time had 14 levels (CT1.0, CT1.24, CT1.48, CT2.0, CT2.24, CT2.48, CT3.0, CT3.24, CT3.48, C.0, C.24, C.48, C.72 and C.96). Factor values were determined on the basis of CT treatments (CT1, CT2, CT3) with the time after exposure (0, 24 or 48 h) and controls with times 0, 24, 48, 72 and 96.

When analysis of variance showed statistical significance (Supplementary Tables S1, S2, S3, S4, S5 and S6), the contrast analysis was performed using the *glht* function with user-defined contrasts from the R package multcomp with a generalized hypothesis testing procedure, considering simultaneous hypothesis tests. The contrasts between the mean values of the CT.time variables were compared to the control within the same time. Where the *P*-value for differences between means was less than 0.05, the difference was considered statistically significant.

## Results

### Chloroplast pigments

The chloroplast pigments detected in sweet cherry leaves were chlorophylls and carotenoids. The average chlorophyll a/b ratio of ‘Grace Star’ was higher (2.23 ± 0.04) than of ‘Schneiders’ (2.00 ± 0.03). The predominant carotenoids were lutein and β-carotene, which accounted for more than 60%, while the rest consisted of neoxanthin, violaxanthin, antheraxanthin and zeaxanthin. Violaxanthin showed to be the main pigment of the xanthophyll cycle (more than 76% on average) in sweet cherry leaves, while antheraxanthin (in Experiment I) together with zeaxanthin (in Experiment II) made up the rest.

**Figure 2:**
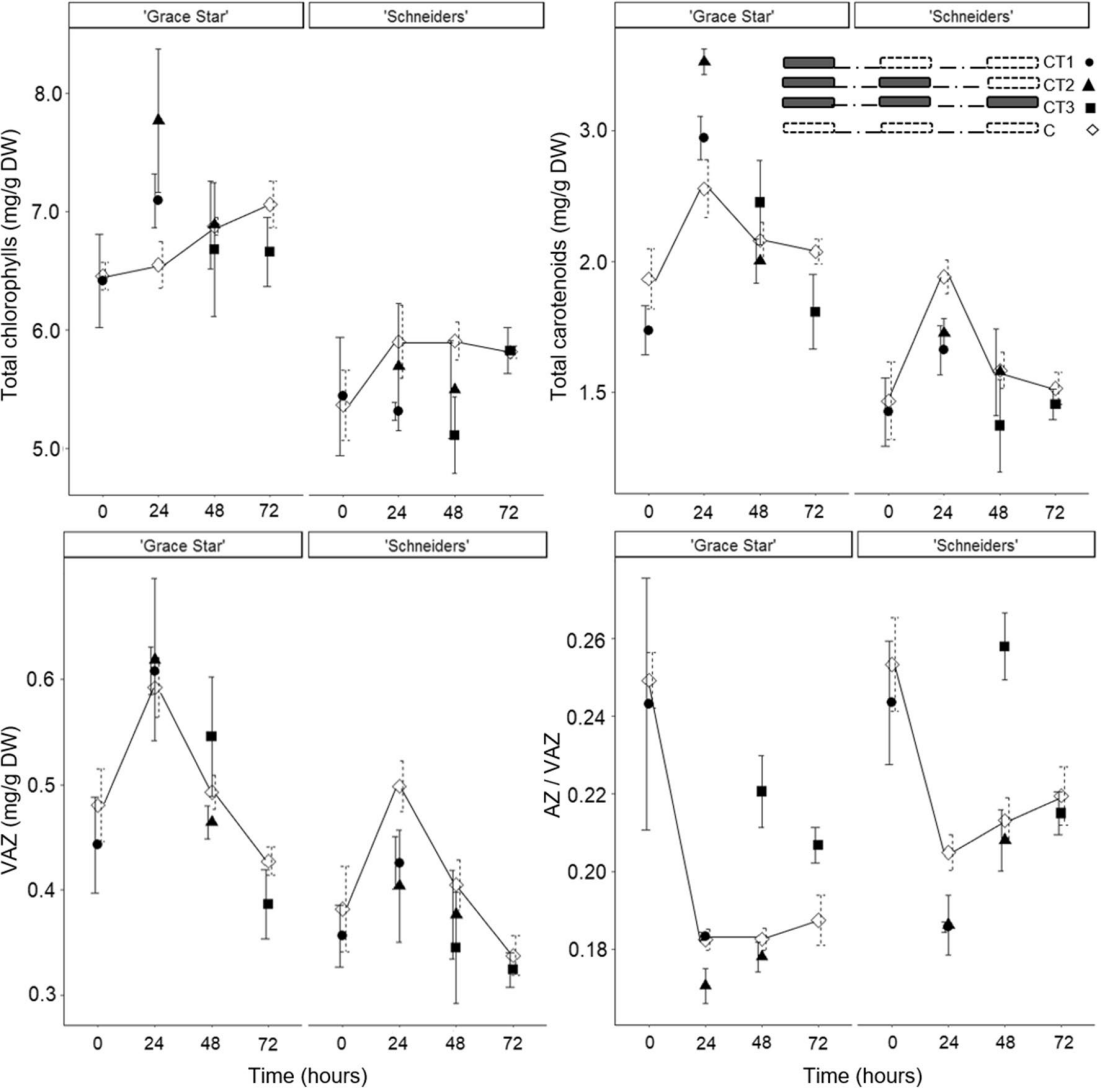
The total chlorophylls, total carotenoids, VAZ (xanthophyll cycle pigments) and AZ/VAZ (deepoxidation state of xanthophyll cycle pool) in leaves of ‘Grace Star’ and ‘Schneiders’, sampled at time 0, time 24, time 48 and time 72 in Experiment I. The overnight chilling treatment (CT) comprises four patterns: CT1 (one exposure, black filled circle); CT2 (two exposures, black filled triangle); CT3 (three exposures, black filled square) and C (control trees, white filled diamond). A black filled rectangle indicates the night-time exposure to CT, while white filled rectangle indicates the night under natural conditions. Vertical bars represent ± SE of the mean (n = 3; n = 3 and 6 for controls).

In Experiment I, 'Grace Star' showed a statistically significant higher average content of all chloroplast pigments than 'Schneiders' (Fig. 2). The factor CT.time had a statistically significant influence on chlorophyll b, chlorophyll a/b ratio, all detected carotenoids, total carotenoids, VAZ and AZ/VAZ (Supplementary Table S1). A further contrast analysis showed no statistically significant differences between the CT.time levels and the corresponding controls, with the exception of CT3. The leaves of CT3.0 trees showed a statistically significant higher AZ/VAZ ratio compared to the control (Table 1).

**Table 1:** The results of contrast analysis of selected biochemical and physiological parameters in sweet cherry leaves exposed to one (CT1), two (CT2) or three (CT3) overnight chillings, sampled at time 0 or time 24 and compared with control in Experiment I.

	CT1		CT2		CT3	
	0	24	0	24	0	24
Chlorophyll a	ns	ns	ns	ns	ns	ns
Chlorophyll b	ns	ns	ns	ns	ns	ns
Chlorophyll a/b	ns	ns	ns	ns	ns	ns
Total chlorophylls	ns	ns	ns	ns	ns	ns
β-carotene	ns	ns	ns	ns	ns	ns
Lutein	ns	ns	ns	ns	ns	ns
Neoxanthin	ns	ns	ns	ns	ns	ns
Violaxanthin	ns	ns	ns	ns	ns	ns
Antheraxanthin	ns	ns	ns	ns	ns	ns
Zeaxanthin	ns	ns	ns	ns	ns	ns
Total carotenoids	ns	ns	ns	ns	ns	ns
VAZ	ns	ns	ns	ns	ns	ns
AZ/VAZ	ns	ns	ns	ns	↑***	ns
Fv/Fm	ns	ns	ns	ns	–	–

*Statistically significant differences at *P* < 0.05; ***statistically significant differences at *P* < 0.001; ns, not significant; ↑ = increase in chilled leaves; ↓ = decrease in chilled leaves; –, not monitored; VAZ, xanthophyll cycle pigments; AZ/VAZ, deepoxidation state of xanthophyll cycle pool; Fv/Fm, maximum quantum yield of PS II.

In the Experiment II, the factor CT.time had a statistically significant influence on the chlorophyll a/b ratio and the average content of all identified carotenoids except lutein, while there was no influence on chlorophylls (Supplementary Table S3). The content of antheraxanthin, violaxanthin, zeaxanthin, neoxanthin, VAZ and AZ/VAZ ratio changed statistically significantly after CT (chilling treatment). Among the VAZ, zeaxanthin was the most affected and its increase was observed after CT.

Sweet cherry leaves of CT1.0, CT2.0 and CT3.0 trees had a statistically significantly higher average zeaxanthin content and a 1.9-, 2.3- and 2.1-fold, respectively, higher AZ/VAZ ratio than the corresponding control. The leaves of CT2.0 trees had a statistically significantly lower average content of neoxanthin and violaxanthin than control. The leaves of CT3.0 trees had a statistically significantly higher content of antheraxanthin and VAZ than the control.

**Figure 3:**
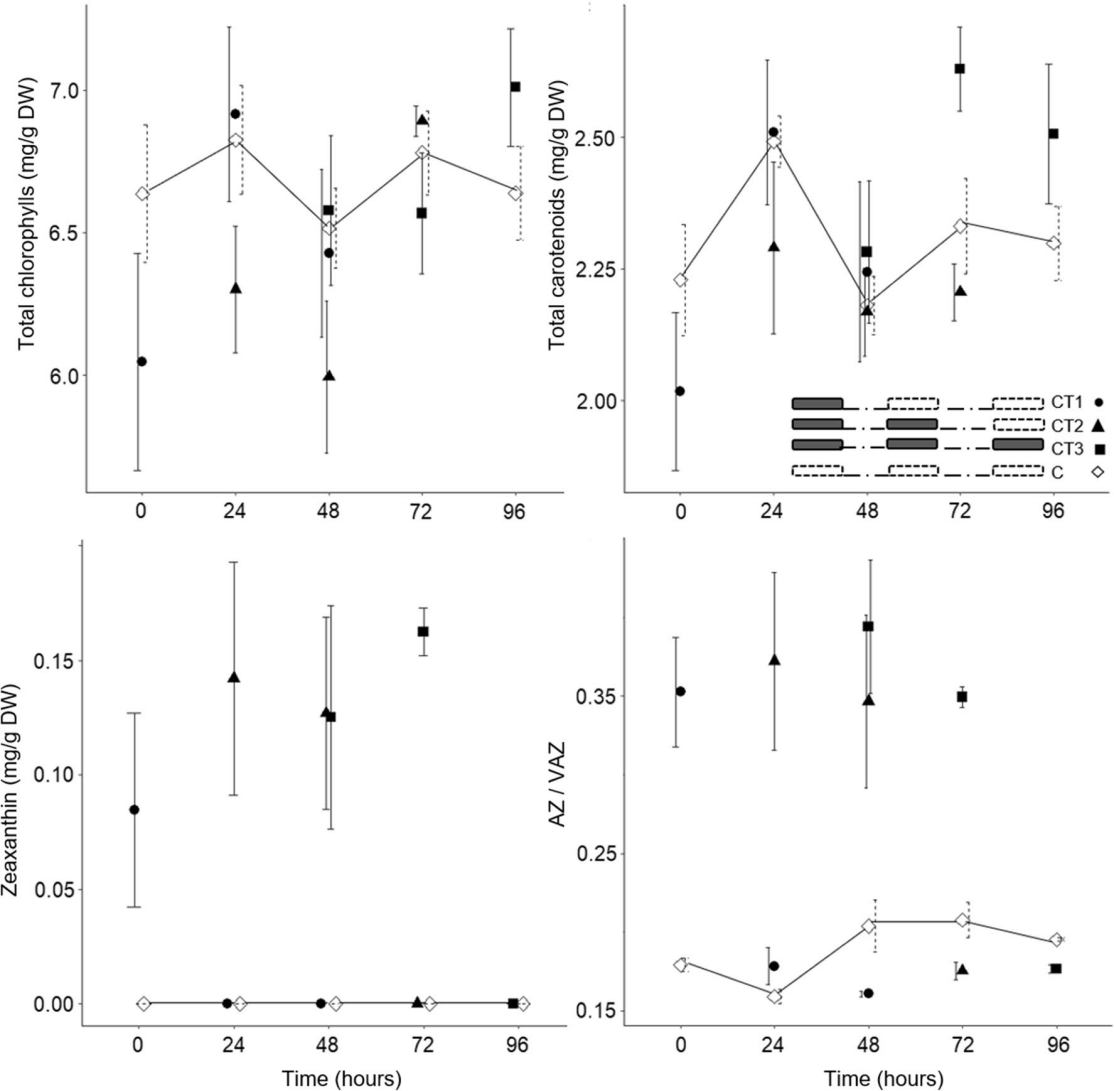
The total chlorophylls, total carotenoids, zeaxanthin content and AZ/VAZ (deepoxidation state of xanthophyll cycle pool) in leaves of ‘Grace Star’ sampled at time 0, time 24, time 48, time 72 and time 96 in Experiment II. Vertical bars represent ± SE of the mean (n = 3; n = 3, 6 or 9 for controls). For other bar details, see Fig. 2.

Thereafter, the sweet cherry leaves recovered differently. Zeaxanthin in the leaves of CT1 trees diminished within 24 h, whereas it diminished in the leaves of CT2 and CT3 trees within 48 h (Fig. 3). The leaves of CT2.24 and CT3.24 trees showed a statistically significantly higher average zeaxanthin and AZ/VAZ ratio than the corresponding controls (Table 2, Fig. 3). In connection with the complete reduction of zeaxanthin, the leaves of CT1.24, CT2.48 and CT3.48 trees showed a similar AZ/VAZ ratio than the corresponding controls (Table 2, Fig. 3).

**Table 2:** The results of the contrast analysis of the selected biochemical and physiological parameters in sweet cherry leaves exposed to one (CT1), two (CT2) or three (CT3) overnight chillings, sampled at time 0, 24 and 48 and compared to control in Experiment II.

	CT1			CT2			CT3		
	0	24	48	0	24	48	0	24	48
Chlorophyll a	ns	ns	ns	ns	ns	ns	ns	ns	ns
Chlorophyll b	ns	ns	ns	ns	ns	ns	ns	ns	ns
Chlorophyll a/b	ns	ns	ns	ns	ns	ns	ns	ns	ns
Total chlorophylls	ns	ns	ns	ns	ns	ns	ns	ns	ns
β-carotene	ns	ns	ns	ns	ns	ns	ns	ns	ns
Lutein	ns	ns	ns	ns	ns	ns	ns	ns	ns
Neoxanthin	ns	ns	ns	↓*	ns	ns	ns	ns	ns
Violaxanthin	ns	ns	ns	↓*	ns	ns	ns	ns	ns
Antheraxanthin	ns	ns	ns	ns	ns	ns	↑*	ns	ns
Zeaxanthin	↑*	ns	ns	↑***	↑***	ns	↑***	↑***	ns
Total carotenoids	ns	ns	ns	ns	ns	ns	ns	ns	ns
VAZ	ns	ns	ns	ns	ns	ns	↑*	↑***	ns
AZ/VAZ	↑***	ns	ns	↑***	↑***	ns	↑***	↑***	ns
Glucose	ns	ns	ns	ns	ns	ns	ns	ns	ns
Fructose	ns	ns	ns	ns	ns	ns	ns	ns	↑**
Sucrose	ns	↑***	ns	ns	ns	↑*	ns	↑**	↑***
Sorbitol	ns	ns	↑*	ns	ns	↑**	ns	↑***	↑**
Total sugars	ns	ns	ns	ns	ns	↑*	ns	↑**	↑**
Neochlorogenic acid	ns	ns	ns	ns	ns	ns	↑**	ns	ns
Chlorogenic acid	ns	ns	ns	ns	ns	ns	ns	ns	ns
3,5-di-*O*-caffeoylquinic acid	ns	ns	ns	ns	ns	ns	ns	ns	ns
Total hydroxycinnamic acids	ns	ns	ns	ns	ns	ns	ns	ns	ns
Catechin	ns	ns	ns	ns	ns	ns	ns	ns	ns
Epicatechin	ns	ns	ns	ns	↑**	ns	ns	ns	ns
Quercetin-3-*O*-rutinoside	ns	ns	ns	ns	ns	ns	ns	ns	ns
Quercetin-3-*O*-glucoside	ns	ns	ns	ns	ns	ns	ns	ns	ns
Quercetin-diglucoside	ns	ns	ns	ns	ns	ns	ns	ns	ns
Kaempferol-3-*O*-glucoside	ns	ns	ns	ns	ns	ns	ns	ns	ns
Total flavonoids	ns	↑*	ns	↑*	ns	ns	ns	ns	↑*
Fv/Fm	↓***	ns	ns	↓***	↓***	ns	↓***	↓***	↓***

*Statistically significant differences at *P* < 0.05; **statistically significant differences at *P* < 0.01; ***, statistically significant differences at *P* < 0.001; ns, not significant. ↑ = increase in chilled leaves; ↓ = decrease in chilled leaves; –, not monitored. VAZ, xanthophyll cycle pigments; AZ/VAZ, deepoxidation state of xanthophyll cycle pool; Fv/Fm, maximum quantum yield of PS II.

### Sugars

**Figure 4:**
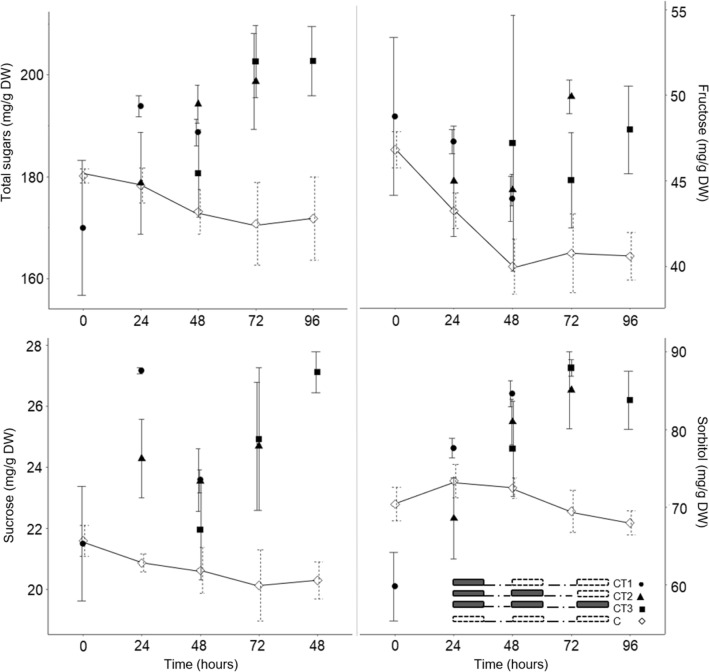
The total sugars, fructose, sucrose and sorbitol content in leaves of ‘Grace Star’ sampled at time 0, time 24, time 48, time 72 and time 96 in Experiment II. Vertical bars represent ± SE of the mean (n = 3; n = 3, 6 or 9 for controls). For other bar details, see Fig. 2.

The results for sugars are shown in Fig. 4 and Table 2. The sugars identified in sweet cherry leaves were glucose, fructose, sucrose and sorbitol. Sorbitol made up the largest part of the total sugar content measured in sweet cherry leaves, while sucrose represented the smallest part.

The analysis of variance showed a statistically significant impact of CT.time on the average total sugars (*P* < 0.001), sucrose (*P* < 0.001), fructose (*P* < 0.05) and sorbitol (*P* < 0.001) (Supplementary Table S5). The glucose content was in the range of 33.9–45.2 mg/g DW and was not significantly influenced by CT (Table 2).

No differences between CT and controls were observed at time 0. Average sugar content increased within 24 and 48 h after CT. The leaves of CT1.24 and CT3.24 trees had a statistically significantly higher average sucrose content, while the leaves of CT3.24 trees also had a statistically significantly higher average total sugar and sorbitol content than the control (Table 2, Fig. 4). Significant changes were observed in the leaves of CT1.48, CT2.48 and CT3.48 trees, where sorbitol content was statistically significantly higher than in the corresponding control. The leaves of CT2.48 trees also had a statistically significantly higher average total sugar content, while leaves of CT3.48 trees additionally had a higher average fructose, sucrose and total sugar content than the corresponding controls (Table 2, Fig. 4).

### Phenolic compounds

Among the phenolic compounds, two main groups were identified in the sweet cherry leaves: hydroxycinnamic acids (neochlorogenic acid, chlorogenic acid and 3,5-di-*O*-caffeoylquinic acid) and flavonoids (catechin, epicatechin, quercetin-3-*O*-rutinoside, quercetin-3-*O*-glucoside, kaempferol-3-*O*-glucoside and quercetin-diglucoside). The quantitative analysis of the phenolic compounds in the leaves of the sweet cherry showed that chlorogenic acid was the main identified hydroxycinnamic acid. Among the identified flavonoids, catechin and quercetin-3-*O*-rutinoside represented the majority.

**Figure 5:**
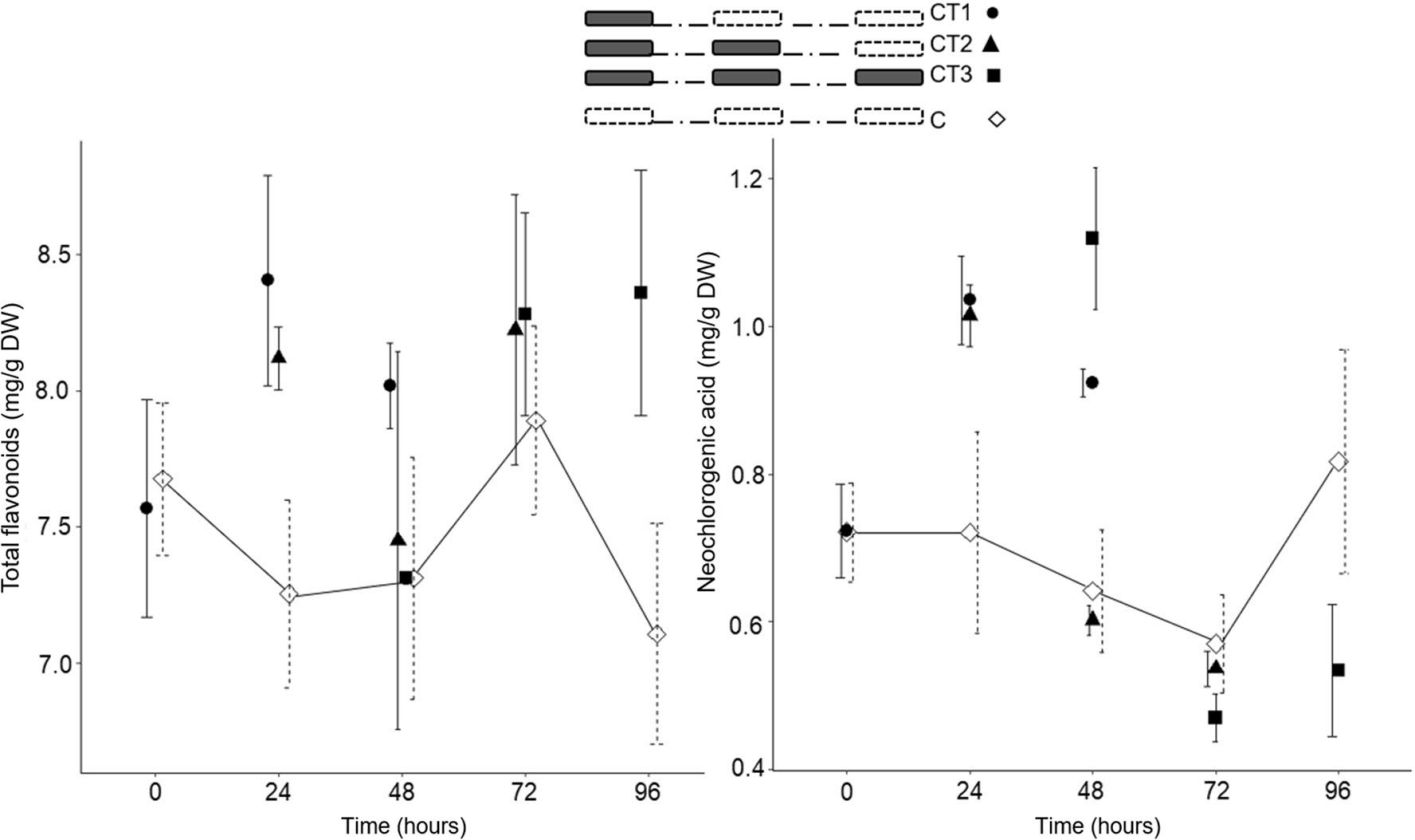
The content of total flavonoids and neochlorogenic acid in leaves of ‘Grace Star’ sampled at time 0, time 24, time 48, time 72 and time 96 in Experiment II. Vertical bars represent ± SE of the mean (n = 3; n = 3, 6 or 9 for controls). For other bar details, see Fig. 2.

The analysis of variance showed a statistically significant impact of CT.time on the content of neochlorogenic acid, chlorogenic acid, epicatechin and total flavonoids (Supplementary Table S6). Further contrast analysis showed that the average content of neochlorogenic acid, epicatechin, and total flavonoids in the leaves of CT trees differed from the controls (Table 2). Leaves of CT3.0 trees had a statistically significantly higher average content of neochlorogenic acid than the control. The leaves of CT2.24 trees had a statistically significantly higher average content of epicatechin than the control. The average content of total flavonoids was statistically significantly higher in leaves of CT1.24, CT2.0 and CT3.48 trees than in the corresponding controls (Table 2 and Fig. 5).

## Discussion

In our study, the responses of sweet cherry trees to chilling after flowering were monitored by analyzing chloroplast pigments, sugars and phenolic compounds in the leaves. During chilling, zeaxanthin among the carotenoids, sucrose and sorbitol among the sugars and total flavonoids among the phenolic compounds were most affected.

Chilling had no effect on the content of chlorophylls analyzed in sweet cherry leaves. In accordance with our results, some other authors did not observe changes in chlorophyll content in basil plants^33^ and maize^34^ exposed at 6 °C for 8 and 10 days, respectively. In contrast, a reduction in leaf chlorophyll content was observed in *Vitis vinifera* exposed at 5 °C for 7 days^7^ and coffee plants exposed at 4 °C for 3 days^23^. There are no reports of chilling-tolerant fruit species, such as sweet cherry. Chilling can usually affect chlorophyll biosynthesis in plants, as several chlorophyll biosynthesis enzymes are affected and chloroplasts develop slowly^35^. The absence of differences in leaf chlorophyll content in our study shows, that the protective pigments and other antioxidants in the leaves were in all likelihood effective enough to protect the chlorophylls from decay.

The content of xanthophyll cycle pigments in the leaves of the sweet cherry, which were exposed to chilling at 2.2 °C, increased whereas no differences were observed after chilling at 4.7 °C. A significant increase of zeaxanthin was observed. Chilling most likely stimulated the biosynthesis of zeaxanthin due to an increased synthesis of xanthophyll cycle pigments and increased deepoxidation to dissipate excess energy. Zeaxanthin is formed from violaxanthin via antheraxanthin by enzymatic deepoxidation in the xanthophyll cycle^36^. Similarly, some other authors reported increased zeaxanthin content as a result of chilling^23,28^. Xanthophylls are known protective substances that act by thermal dissipation, which avoids energy excitation and reduces the formation of ROS during chilling^37^. As expected, the reduction of violaxanthin was high in our study. Most of the violaxanthin seems to be enzymatically converted by the deepoxidation to antheraxanthin and zeaxanthin through the xanthophyll cycle in sweet cherry leaves exposed to chilling at 2.2 °C. It has been shown that *Zea mays* reacts with changes in leaf pigment composition when exposed to low temperature (14 °C for 5 days) with an accumulation of deepoxidized zeaxanthin and antheraxanthin^28^, but there is no data on chilling-tolerant fruit species. An accumulation of zeaxanthin has also been reported in response to drought stress^25^.

The accumulation of zeaxanthin in our study changed the deepoxidation state ratio of xanthophyll cycle pigments (AZ/VAZ), which increased after chilling at 2.2 °C. AZ/VAZ is known as the parameter expressing the capacity to dissipate the absorbed excitation energy as heat^11^, which is accumulated due to stress conditions and cannot be used for photosynthesis or dissipated in any other way. These results are consistent with the observations of Haldimann^28^. Xanthophyll pigments play an important role in the plant protection mechanism^38^. Through the xanthophyll cycle, these pigments enable thermal, non-photochemical dissipation of excess energy, and protect the photosystem II (PSII) from damage in plants exposed to chilling^36,37^.

Afterwards, the leaves of sweet cherry trees recovered differently, depending on the chilling treatments (CT). The recovery period, during which zeaxanthin decreased, differed between one and several overnight CT. Trees exposed to chilling once diminished the leaf zeaxanthin content within 24 h, i.e. fully epoxidation via the xanthophyll cycle occurred and the AZ/VAZ ratio was reduced. Meanwhile, after several CT (CT2 and CT3) at 2.2 °C, zeaxanthin was fully epoxidized within 48 h. The epoxidation rate of zeaxanthin in the leaves was slower after several CT, probably due to depression of the synthesis or activities of the epoxidase, as reported by Liu et al.^13^. They also showed that AZ/VAZ of *Capsicum annuum* L. exposed at 4 °C for 8 h recovered within 10 h.

Individual sugars identified in the sweet cherry leaf were already determined by Michailidis et al.^39^ and Ranney et al.^40^. In this study an accumulation of sucrose, glucose, sorbitol and fructose in sweet cherry leaves was observed within 24 and 48 h after CT. Higher total sugar content under chilling was mainly attributed to higher sucrose and sorbitol and partly to fructose. Our results are consistent with Miao et al.^12^ and Mitchell and Madore^41^, who reported increased sugar content in *Cucumis sativus* after overnight exposure at 12 °C and in *Cucumis melo* after exposure at 10 °C for 72 h. There are no data on the response of the leaf sugar of tolerant fruit species to chilling temperature exposure.

One CT induced accumulation of only sucrose (after 24 h) and sorbitol (after 48 h). Meanwhile, several CT induced greater accumulation of sucrose, fructose, sorbitol and total sugar content within 24 and 48 h. The accumulation of sugars due to the chilling can be explained by the activation of specific enzymes involved in the biosynthetic pathway. This suggests that although chilling inhibits sucrose synthesis and photosynthesis, various biochemical and physiological stress mechanisms counteract these effects^16^. In accordance with our results, Renaut et al.^6^ reported the highest content of glucose, fructose and sucrose in poplar leaves after 2 days of chilling (4 °C) compared to control. Popov et al.^42^ also reported that the total sugar content in tobacco and Arabidopsis leaves increased by 30 and 60%, respectively, on the sixth day of low temperature exposure (8 and 2 °C), mainly due to the increase in sucrose.

The recording of sugars, an end product of photosynthesis, especially sucrose, which plays a key role^43^, shows that plant metabolism reacts to certain environmental situations^44^. The results of our study also showed a reduced Fv/Fm in the leaves of trees exposed to chilling (Table 2), suggesting that the leaves of sweet cherry trees in our study suffered stress due to exposure to CT in Experiment II. At the same time, the sugar content increased. In general, an excess of sugars triggers the repression of genes associated with photosynthesis and thus an inhibition of photosynthesis, whereas a low sugar content promotes photosynthesis^44^. The accumulation of sugars in stressed leaves could cause a modulation of photosynthesis through a negative feedback mechanism^45^. Sugars could maintain a balance between the rates of CO_2_ assimilation and the electrons supplied by the photosynthetic electron transport chain^43^. It has also been reported that the accumulation of sugars may be associated with increased atmospheric CO_2_ concentration, nitrogen-deficiency and/or changes in phloem loading/unloading for the transport of sugars under different stress conditions^44^. Our results confirmed that the CT induced cell disorders. Chilling may provoke an imbalance between the energy absorbed by the source and the energy consumed by the metabolic sinks, leading to the formation of ROS in PSI and PSII and over reduction of the electron transport chain in the thylakoid membranes. Sugars mitigate the response to chilling stress by acting as compatible solutes and can also act as scavengers of ROS^46^. Similarly, in the case of drought stress, the accumulation of sugars, especially sorbitol, in the leaves of various fruit crops, such as *Malus domestica*^25^, *Prunus persica*^47^, *Prunus avium* × *pseudocerasus* and *Prunus cerasus*^40^ has been reported.

As for the leaf phenolic compounds identified in our study, they have already been identified by Oszmiański and Wojdyło^48^ in *Prunus cerasus* leaf and by Jesus et al.^49^ in *Prunus avium* leaf. Our study showed that among the identified phenolics, chilling affected the content of neochlorogenic acid, epicatechin and total flavonoids. Phenolics are widely distributed secondary metabolites with antioxidant properties in plants, whose biosynthesis and accumulation is associated with their response to stress stimuli^50^. Several chilling in our study increased neochlorogenic acid and epicatechin, while both, one and several CT increased total flavonoids in the sweet cherry leaves. The total flavonoid content was most affected by several CT with the increase occurred after the second chilling and within 48 h after the third CT. A higher phenolic content as a result of chilling, could be due to increased activity of the phenylalanine ammonia lyase (PAL) enzyme^50^. Rivero et al.^50^ found significant increases in soluble phenolics and the highest phenylalanine ammonia-lyase activity in *Citrullus lanatus* plants exposed to chilling (15 °C) for 30 days. PAL is the first enzyme of the phenylpropanoid pathway, essential for the biosynthesis of phenolic acids and flavonoids, which play an important role in the plant’s defense against stress^51^.

Under chilling, plants accumulate several secondary metabolites, especially flavonoids, which provide antioxidant protection against ROS, by localizing and neutralizing free radicals and ROS before they damage the cell^14^ and can inhibit lipid peroxidation, due to their strong ability to donate electrons and hydrogen atoms^52^. Lee and Oh^53^ reported a higher total phenolic content in *Brassica oleracea* var. *acephala* 1 day after exposure to 4 °C. Previous studies have shown that chilling-induced stress affected phenolic compounds in pea roots^54^, soybean roots^55^ and winter wheat leaves^56^, while there are no reports on the phenolic response to chilling in tolerant fruit species.

Chilling caused disturbances in leaf biochemistry in our study, which may affect further fruit development^9^. It is already known that abiotic stress leads to an imbalance between source and sink organs, which can result in flower and fruit drop and thus yield loss^57^. Yield varies from year to year and cultivar to cultivar and this has already been identified as a problem in cherry production^8^. Fruit development has also been found to be related to assimilate supply, but the exact mechanisms are far from fully understood^57^. Given the increasing threat of chilling temperatures in early spring, it is also of great importance for breeders to understand the molecular responses of plants to chilling stress and to develop new molecular approaches to improve chilling tolerance of sweet cherry, as has been shown for other abiotic stresses^58^.

In conclusion, the exposure of sweet cherry trees to chilling after flowering has influenced the biochemical status of the leaves. It can be assumed that a temperature of 2.2 °C induces chilling stress. This was most likely due to a greater deviation of the chilling temperature from the base temperature for sweet cherry growth, which is 4.5 °C. When plants in temperate climates are exposed to the cold in spring, they activate various biochemical changes to alleviate the stress conditions. The diversity and extent of the biochemical changes can vary depending on the intensity and duration of the chilling. In this way, research provides information on an effect of the less studied reaction of sweet cherry trees to chilling temperatures after flowering in spring.

## Supplementary Information

Supplementary Information 1.

## Abbreviations

V: Violaxanthin
A: Antheraxanthin
Z: Zeaxanthin
VAZ: Xanthophyll cycle pigments
AZ/VAZ: Deepoxidation state of xanthophyll cycle pool
ROS: Reactive oxygen species
h: Hour
PSI: Photosystem I
PSII: Photosystem II
Fv/Fm: Maximum quantum yield of PSII
SE: Standard error
CT: Chilling treatment
CT1: One exposure to chilling
CT2: Two consecutive exposures to chilling
CT3: Three consecutive exposures to chilling
CT1.0: Sampling immediately after one chilling
CT2.0: Sampling immediately after two consecutive chilling
CT3.0: Sampling immediately after three consecutive chilling
CT1.24: Sampling 24 h after one chilling
CT2.24: Sampling 24 h after two consecutive chilling
CT3.24: Sampling 24 h after three consecutive chilling
CT1.48: Sampling 48 h after one chilling
CT2.48: Samplings 48 h after two consecutive chilling
CT3.48: Sampling 48 h after three consecutive chilling
C.0: Sampling of controls at time 0
C.24: Sampling of controls at time 24
C.48: Sampling of controls at time 48
C.72: Sampling of controls at time 72
C.96: Sampling of controls at time 96
