# Supplementary material for: Response of chloroplast pigments, sugars and phenolics of sweet cherry leaves to chilling
Source: Sci Rep. 2021 Mar 30;11:7210. doi: 10.1038/s41598-021-86732-y (PMC8009888; doi:10.1038/s41598-021-86732-y)
Supplement: Supplementary file 1 — Supplementary Information 1. [file 41598_2021_86732_MOESM1_ESM.docx]

**Response of chloroplast pigments, sugars and phenolics of sweet cherry leaves to chilling**

Matej Vosnjak,^a^* Helena Sircelj, ^b^ Metka Hudina, ^a^ Valentina Usenik ^a^

^a^ University of Ljubljana, Biotechnical Faculty, Department of Agronomy, Chair for Fruit Growing, Viticulture and Vegetable Growing, Jamnikarjeva 101, SI-1000 Ljubljana, Slovenia (matej.vosnjak@bf.uni-lj.si, metka.hudina@bf.uni-lj.si, valentina.usenik@bf.uni-lj.si)

^b^ University of Ljubljana, Biotechnical Faculty, Department of Agronomy, Chair of Applied Botany, Plant Ecology & Physiology and Informatics, Jamnikarjeva 101, SI-1000 Ljubljana, Slovenia (helena.sircelj@bf.uni-lj.si)

*Corresponding author:

Matej Vosnjak

+386 1 320 3143

matej.vosnjak@bf.uni-lj.si

SUPPLEMENTARY TABLES

Supplementary Table S1: Statistically significant differences (ANOVA) for main factors and their interaction of chloroplast pigments in Experiment I.

|  | df | chlA | chlB | chl a/b | Tchl | lut | betkar | neo | antera | viola | Tcar | VAZ | AZ/VAZ |
| --- | --- | --- | --- | --- | --- | --- | --- | --- | --- | --- | --- | --- | --- |
| Cultivar | 1 | *** | *** | *** | *** | *** | *** | *** | *** | *** | *** | *** | *** |
| CT.time | 9 | ns | * | *** | ns | *** | *** | ** | *** | *** | *** | *** | *** |
| Cultivar:CT.time |  | ns | ns | ns | ns | ns | ns | ns | ns | ns | ns | ns | ns |

ANOVA: *, statistically significant differences at P < 0.05; **, statistically significant differences at P < 0.01; ***, statistically significant differences at P < 0.001; ns, not significant; df, degrees of freedom.

Chlorophyll a (chlA), chlorophyll b (chlB), chlorophyll a/b ratio (chl a/b), lutein (lut), β-carotene (betkar), violaxanthin (viola), neoxanthin (neo), antheraxanthin (antera), zeaxanthin (zea), xantophyll cycle pigments (VAZ), deepoxidation state of xanthophyll cycle pool (AZ/VAZ), total chlorophylls (Tchl), total carotenoids (Tcar).

Supplementary Table S2: Statistically significant differences (ANOVA) for main factors and their interaction of maximum quantum yield of PS II at time 24 and 72 in Experiment I.

|  | df | Fv/Fm |
| --- | --- | --- |
| Cultivar | 1 | ns |
| CT.time | 4 | *** |
| Cultivar:CTE.time |  | ns |

ANOVA: *, statistically significant differences at P < 0.05; **, statistically significant differences at P < 0.01; ***, statistically significant differences at P < 0.001. ns, not significant; df, degrees of freedom; Fv/Fm, maximum quantum yield of PS II.

Supplementary Table S3: Statistically significant differences (ANOVA) for factor CT.time of chloroplast pigments in Experiment II.

|  | df | chlA | chlB | chl a/b | Tchl | lut | betkar | neo | antera | viola | zea | Tcar | VAZ | AZ/VAZ |
| --- | --- | --- | --- | --- | --- | --- | --- | --- | --- | --- | --- | --- | --- | --- |
| CT.time | 13 | ns | ns | ** | ns | ns | *** | ** | ** | *** | *** | * | *** | *** |

ANOVA: *, statistically significant differences at P < 0.05; **, statistically significant differences at P < 0.01; ***, statistically significant differences at P < 0.001; ns, not significant; df, degrees of freedom.

Chlorophyll a (chlA), chlorophyll b (chlB), chlorophyll a/b ratio (chl a/b), lutein (lut), β-carotene (betkar), violaxanthin (viola), neoxanthin (neo), antheraxanthin (antera), zeaxanthin (zea), xantophyll cycle pigments (VAZ), deepoxidation state of xanthophyll cycle pool (AZ/VAZ), total chlorophylls (Tchl), total carotenoids (Tcar).

Supplementary Table S4: Statistically significant differences (ANOVA) for main factor of maximum quantum yield of PS II at times 0, 24 and 48 in Experiment II.

|  | df | Fv/Fm |
| --- | --- | --- |
| CT.time | 14 | *** |

ANOVA: *, statistically significant differences at P < 0.05; **, statistically significant differences at P < 0.01; ***, statistically significant differences at P < 0.001; ns, not significant; df, degrees of freedom; Fv/Fm, maximum quantum yield of PS II.

Supplementary Table S5: Statistically significant differences (ANOVA) for main factor of individual and total sugar content in Experiment II.

|  | df | Sucrose | Glucose | Fructose | Sorbitol | Total sugars |
| --- | --- | --- | --- | --- | --- | --- |
| CT:time | 13 | *** | ns | * | *** | *** |

ANOVA: *, statistically significant differences at P < 0.05; **, statistically significant differences at P < 0.01; ***, statistically significant differences at P < 0.001; ns, not significant; df, degrees of freedom.

Supplementary Table S6: Statistically significant differences (ANOVA) for main factor of individual identified phenolic compounds and main groups in Experiment II.

|  | df | neoa | chla | caffq | HCA | cat | epi | qrut | qglu | kglu | qdiglu | Total flavonoids |
| --- | --- | --- | --- | --- | --- | --- | --- | --- | --- | --- | --- | --- |
| CT.time | 13 | *** | * | ns | ns | ns | *** | ns | ns | ns | ns | * |

ANOVA: *, statistically significant differences at P < 0.05; **, statistically significant differences at P < 0.01; ***, statistically significant differences at P < 0.001; ns, not significant; df, degrees of freedom. Neochlorogenic acid (neoa), 3,5-di-O-caffeoylquinic acid (caffq), chlorogenic acid (chla), total hydroxycinnamic acids (HCA), catechin (cat), epicatechin (epi), quercetin- 3-O-rutinoside (qrut), quercetin- 3-O-glucoside (qglu), kaempferol-3-O-glucoside (kglu), quercetin-diglucoside (qdiglu).
